# Genetic Factors for Enhancement of Nicotine Levels in Cultivated Tobacco

**DOI:** 10.1038/srep17360

**Published:** 2015-12-02

**Authors:** Bingwu Wang, Ramsey S. Lewis, Junli Shi, Zhongbang Song, Yulong Gao, Wenzheng Li, Hongxia Chen, Rongda Qu

**Affiliations:** 1 Tobacco Breeding Center, Yunnan Academy of Tobacco Agricultural Sciences, Kunming, Yunnan 650021, China; 2 Crop Science Department, North Carolina State University, Raleigh, NC 27695, USA

## Abstract

Nicotine has practical applications relating to smoking cessation devices and alternative nicotine products. Genetic manipulation for increasing nicotine content in cultivated tobacco (*Nicotiana tabacum* L.) may be of value for industrial purposes, including the possibility of enhancing the efficiency of nicotine extraction. Biotechnological approaches have been evaluated in connection with this objective, but field-based results are few. Here, we report characterization of two genes encoding basic-helix-loop-helix (bHLH) transcription factors (TFs), *NtMYC2a* and *NtMYC2b* from tobacco. Overexpression of *NtMYC2a* increased leaf nicotine levels in T_1_ transgenic lines approximately 2.3-fold in greenhouse-grown plants of tobacco cultivar ‘NC 95′. Subsequent field testing of T_2_ and T_3_ generations of transgenic *NtMYC2a* overexpression lines showed nicotine concentrations were 76% and 58% higher than control lines, respectively. These results demonstrated that the increased nicotine trait was stably inherited to the T_2_ and T_3_ generations, indicating the important role that *NtMYC2a* plays in regulating nicotine accumulation in *N. tabacum* and the great potential of *NtMYC2*a overexpression in tobacco plants for industrial nicotine production. Collected data in this study also indicated a negative feedback inhibition of nicotine biosynthesis. Further enhancement of nicotine accumulation in tobacco leaf may require modification of the processes of nicotine transport and deposition.

Nicotine, as a bioactive natural phytochemical, has practical applications. It is the key component of smoking cessation devices and alternative nicotine products, such as electronic cigarette^1^. Nicotine may also play a role in treatments to ameliorate symptoms from Parkinson’s disease^2^,^3^. Although nicotine can be chemically synthesized, this is not currently a cost-effective approach for producing nicotine relative to chemical extraction from tobacco^4^. Tobacco plants with enhanced capacity to accumulate nicotine might be of value for commercial nicotine extraction operations.

In commercial tobacco cultivars, nicotine represents 90–95% of the total alkaloid pool and is synthesized exclusively in tobacco roots, transported to leaves, and stored in leaf cell vacuoles by a multidrug and toxic compound extrusion (MATE) transporter^5^,^6^. Various factors control nicotine biosynthesis and accumulation, including plant genetics, environment conditions, insect predation, mechanical injury and agronomical management measures, such as topping (decapitation of the apical meristem at the early stage of flowering) and suckering (removing the axillary buds of plants activated by topping)^7^,^8^.

Nicotine has two ring moieties, a pyrrolidine ring and a pyridine ring, derived from two branch pathways (Supplementary Fig. S1). The pyrrolidine ring is formed from arginine or ornithine via putrescine and methylputrescine. The first committed enzyme, putrescine methyltransferase (PMT), is under more stringent control than other enzymes of this branch pathway^9^,^10^. There are several other enzymes involved in the pyrrolidine branch route: arginine decarboxylase (ADC) which catalyzes the decarboxylation of arginine; ornithine decarboxylase (ODC) which catalyzes the decarboxylation of ornithine; and methylputrescine oxidase (MPO) which catalyzes the deamination of methylputrescine. The pyridine ring is derived from nicotinic acid, an intermediate of the pyridine nucleotide cycle that is regulated by an entry enzyme of the cycle, quinolinic acid phosphoribosyltransferase (QPT)^11^. The nicotine synthase (NS), which catalyzes the final condensation step between nicotinic acid and methylpyrrolinium cation to form nicotine, has not been elucidated, although two candidate genes (*A622* and *NtBBL*) had been identified^10^,^12^.

Several attempts have been made to increase nicotine levels in tobacco plants using biotechnological methods. Because of the central role that PMT plays in nicotine biosynthesis^13^, *NtPMT1a* was the first nicotine synthetic gene to be overexpressed in *N. sylvestris*, a progenitor species of tobacco, under control of the CaMV 35S promoter. Overexpression in greenhouse-grown plants was found to increase *PMT* transcripts levels 4 to 8 fold, with nicotine levels increased by about 40%^14^. In a separate report, overexpression of *NtPMT1a* or *NtQPT2* individually or simultaneously was shown to enhance leaf nicotine levels in T_0_ transgenic presumably greenhouse-grown plants of *N. tabacum* cv. K326^15^. Wang^16^ obtained contradictory results in field-grown tobacco plants, however, and failed to observe increased nicotine contents in transgenic plants overexpressing *NtPMT1a* and/or *NtQPT2*. Recently, genes encoding for transcription factors were shown to positively regulate nicotine levels. NbbHLH1 and NbbHLH2 were the first two MYC2 related transcription factors characterized in the genus *Nicotiana*. Overexpression of either gene significantly increased nicotine levels in *N. benthamiana*, a small wild *Nicotiana* species. However, the reported nicotine level in fresh leaf of *NbbHLH2* overexpression plants was only about 0.025% (0.25 mg/g)^17^, which is roughly ten times lower than nicotine levels typically observed for field-grown tobacco cultivars. In addition, several ERFs (ethylene response factors), another important class of transcription factors, were shown to positively regulate nicotine biosynthesis^18^,^19^, but their effects on enhancement of nicotine content in cultivated tobacco plants have not been demonstrated.

In another approach, overexpression of the allene oxide cyclase gene, a gene controlling a key step in jasmonate formation, was reported to cause a 4.8 fold increase in nicotine content in transgenic greenhouse-grown T_0_ *N. tabacum* cv. Petit Havana^20^, an early-flowering tobacco type with very low leaf number^21^.

Here, we describe two *NtMYC2* genes, *NtMYC2a* and *NtMYC2b*, isolated from a yeast one hybrid library screening using the promoter of *NtQPT2* as bait and demonstrate that overexpression of either gene can effectively increase nicotine accumulation in greenhouse-grown T_0_ and T_1_ generation tobacco plants of a standard tobacco cultivar. We further demonstrate stable transmission of the increased nicotine trait through the T_2_ and T_3_ generations of *NtMYC2a* overexpression lines. Results from field tests of *NtMYC2a* overexpression lines suggests that transgenic overexpression of this transcription factor may be useful as a novel source to increase the nicotine content in cultivated tobacco. In addition, we observed evidence of a negative feedback loop of nicotine to regulate expression of its synthetic genes in tobacco root. Strategies to further increase nicotine levels in tobacco are discussed.

## Results

### Cloning of *NtMYC2a* and *NtMYC2b* genes from tobacco

The yeast one-hybrid technique was used in this study to identify transcription factors that bind to the promoter sequence of *NtQPT2*. A total of 1.6 million yeast clones were screened. Analysis of sequencing data revealed that a single clone carried a cDNA encoding a bHLH transcription factor, but the 5′ terminal sequence was missing. The full length cDNA sequence was obtained using the Rapid Amplification of cDNA Ends (RACE) technique. During the process, another highly homologous bHLH transcription factor was also identified.

The full-lengths of the two cDNAs were found to be 2214 and 2391 bp encoding for proteins of 659 and 658 amino acids, respectively. The two cDNAs shared 95% identity to each other at the nucleotide sequence level, and BLAST analysis to the NCBI database showed these two transcription factors to be 99.9% (1979/1980 nt) and 100% (1977/1977 nt) identical to the coding sequences of *NtMYC2a* (GenBank No. GQ859160) and *NtMYC2b* (GenBank No. GQ859161). The single nucleotide difference in *NtMYC2a* could be caused by error in sequencing or PCR, or by cultivar difference. The two isolated TF genes were thus designated as *NtMYC2a* and *NtMYC2b*.

**Figure 1:**
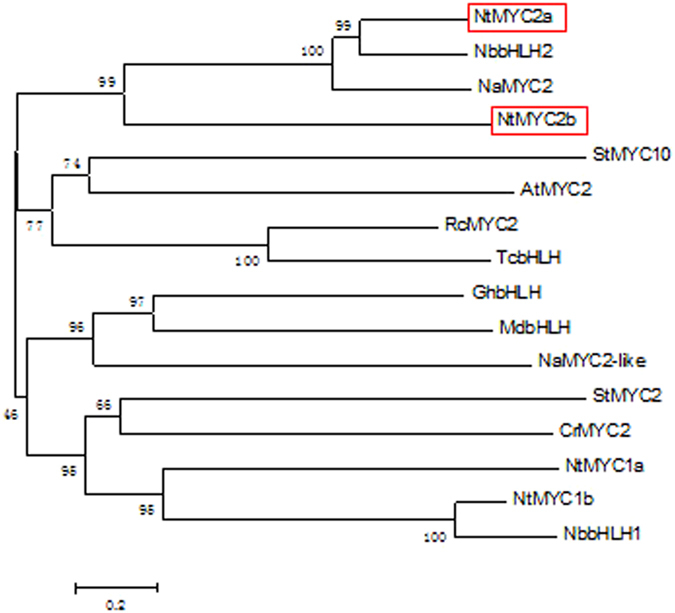
Phylogenetic tree of NtMYC2a, NtMYC2b and 14 other bHLH transcription factors. The tree was constructed using the neighbor-joining method in MEGA5.2 22 with 1000 bootstrap replications. The proteins included in this analysis with accession number in parentheses are: NtMYC2a (ADH04269); NtMYC2b (ADH04270); NaMYC2 (AGL98100); NbbHLH2 (ADH04263); StMYC10 (CAF74711); NtMYC1b (ADH04268); NtMYC1a (ADH04267); NaMYC2-like (AGL98101); StMYC2 (CAF74710); NbbHLH1 (ADH04262); CrMYC2 (AAQ14332); RcMYC2 (XP_002519841); TcbHLH (EOY23994); GhbHLH (ACO53628); MdbHLH (ADL36595); AtMYC2 (NP_174541).

A phylogenetic tree based upon amino acid sequences was constructed using the maximum likelihood method in MEGA5.2 22 to infer the evolutionary relationships of both NtMYC2a and NtMYC2b with other bHLH transcription factors. Several closely related transcription factors from the BLAST result were also included. As expected, NtMYC2a and NtMYC2b are closely related to each other, most closely related to NbbHLH2 and NaMYC2, and more distant to NtMYC1a, NtMYC1b, NbbHLH1 and StMYC2^17^,^23^,^24^,^25^ (Fig. 1).

### *NtMYC2a* and *NtMYC2b* differentially enhanced nicotine levels and affected expression of nicotine synthetic genes

To investigate the effect of the *NtMYC2a* and *NtMYC2b* TF genes on the biosynthesis of nicotine, we evaluated transgenic tobacco plants over-expressing these genes. The coding sequences of *NtMYC2a* and *NtMYC2b* were placed under the control of the CaMV 35S promoter to enhance the expression of the genes. In total, seven *35S:NtMYC2a* and nine *35S:NtMYC2b* T_0_ plants were generated and initially tested.

**Figure 2:**
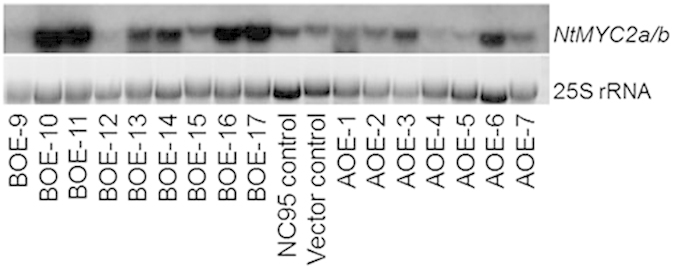
Northern hybridization showing *NtMYC2* mRNA levels in roots of T_0_ transgenic plants before flowering. The bottom panel shows the 25S ribosomal RNA in gel stained with EtBr as an RNA loading reference. AOE: *NtMYC2a* overexpression; BOE: *NtMYC2b* overexpression.

**Figure 3:**
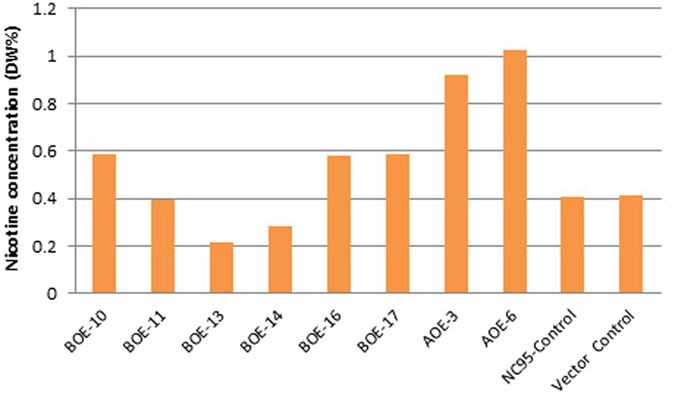
Leaf nicotine levels of T_0_ overexpression individuals. DW%: dry weight percentage. AOE: *NtMYC2a* overexpression; BOE: *NtMYC2b* overexpression.

**Figure 4:**
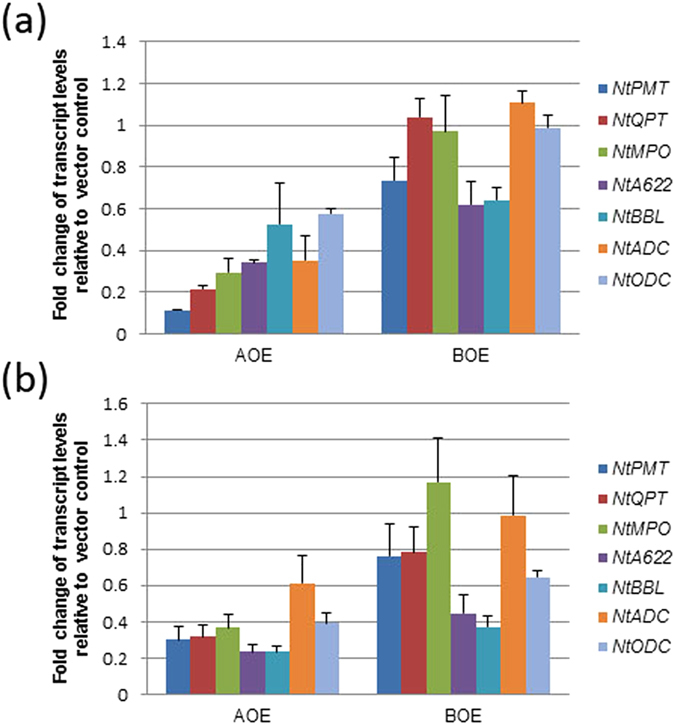
Expression patterns of nicotine synthetic genes measured by qRT-PCR. (**a**) The average expression level of T_0_ plants is shown. Two T_0_ plants from each construct that affects the nicotine level most were analyzed (AOE-3, AOE-6 for *NtMYC2a* and BOE-16, BOE-17 for *NtMYC2b*). (**b**) The average expression levels of nicotine synthetic genes in T_1_ plants are shown. Two transgene positive plants of each T_1_ line and thus total four plants for each construct were analyzed. The expression level in the vector control was set to 1, and expression of nicotine synthetic genes in T_0_ and T_1_ transgenic plants is shown relative to this level, the error bars indicate standard errors. AOE: *NtMYC2a* overexpression; BOE: *NtMYC2b* overexpression.

Northern analysis was performed to test the expression levels of *NtMYC2a/b* in T_0_ greenhouse-grown plants. The results indicated that two *NtMYC2a* transgenic plants (AOE-3 and AOE-6) and six *NtMYC2b* transgenic plants (BOE-10, 11, 13, 14, 16 and 17) clearly exhibited increased expression of these genes compared to the wild type and vector control plants (Fig. 2). Leaf nicotine levels of these plants were quantified. Three *35S:NtMYC2b* plants (BOE-10, 16 and 17) accumulated higher nicotine levels (approximately 40% over the vector control) while the other three plants (BOE-11, 13 and 14) produced nicotine levels that were similar to, or lower than, the controls. Two *35S:NtMYC2a* T_0_ plants (AOE-3 and AOE-6) exhibited much higher nicotine levels as compared to the controls (123% and 150% higher, respectively, than the vector control) (Fig. 3). Based on this initial data, we selected two 35S:*NtMYC2a* and two 35S:*NtMYC2b* overexpression plants (AOE-3, AOE-6, BOE-16 and BOE-17) to study the effects of overexpression of these two TFs on the expression of genes associated with the nicotine biosynthetic pathway using quantitative real-time reverse transcription-PCR (qRT-PCR). Overexpression of *NtMYC2b* did not substantially alter the mRNA levels of the nicotine synthetic genes except for a moderate reduction in the expression of the *NtPMT*, *NtA622* and *NtBBL* genes (Fig. 4a). However, the expression of all the evaluated nicotine synthetic genes was remarkably reduced by overexpression of *NtMYC2a*. We extended the transcript quantification of the seven nicotine synthetic genes to plants of T_1_ generation of those overexpressing lines. Two plants from each line and a total of four plants for each *NtMYC2* gene construct were analyzed. The results largely confirmed observations for the T_0_ plants. The expression of the nicotine synthetic genes was much suppressed in *NtMYC2a* overexpression lines as compared to vector control (Fig. 4b). No obvious growth or developmental differences were noted between the transgenic and wild-type plants.

### Inheritance of high nicotine content in T_1_ *NtMYC2a* and *NtMYC2b* overexpression lines

**Figure 5:**
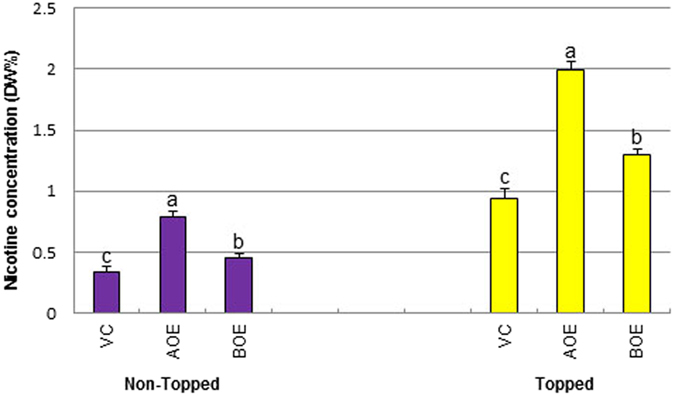
Leaf nicotine levels in T_1_ transgenic plants under non-topping or topping treatment. DW%: dry weight percentage. Values are average of T_1_ transgenic plants (PCR positive, n = 15–30) with standard errors. Different letters indicate significant difference within each category (t-test, *P* < 0.05). VC: Vector Control; AOE: *NtMYC2a* overexpression; BOE: *NtMYC2b* overexpression.

To further confirm the functions of both *NtMYC2* genes and to determine the potential of inheritance of the elevated nicotine trait, T_1_ generation families were produced by self-pollination of AOE-3, AOE-6, BOE-16, BOE-17 and vector control T_0_ plants. T_1_ plants were grown in greenhouse for approximately two months and PCR was used to identify transgenic segregants. Leaf nicotine levels were determined in non-topped plants and plants ten days after topping. The results (Fig. 5) showed that non-topped *NtMYC2a* overexpression (AOE-3 and AOE-6) T_1_ generation plants had nicotine levels that were 134% higher than that of the vector control, while *NtMYC2b* overexpression plants (BOE-16 and BOE-17) exhibited 35% increase in nicotine content. Similar increases were observed in the topping treatment. Results were highly consistent with those observed in the T_0_ plants (Fig. 3), indicating inheritance of high-nicotine trait in these transgenic plants.

In both the topped and non-topped treatments, nicotine levels of *NtMYC2a* overexpression plants were shown to be significantly greater than those of *NtMYC2b* overexpression plants (Student’s t-test, *P* < 0.0001), indicating these two closely-related paralogs function differentially to affect nicotine biosynthesis in tobacco.

### Field performance of T_2_ and T_3_ *NtMYC2* overexpression transgenic lines

**Figure 6:**
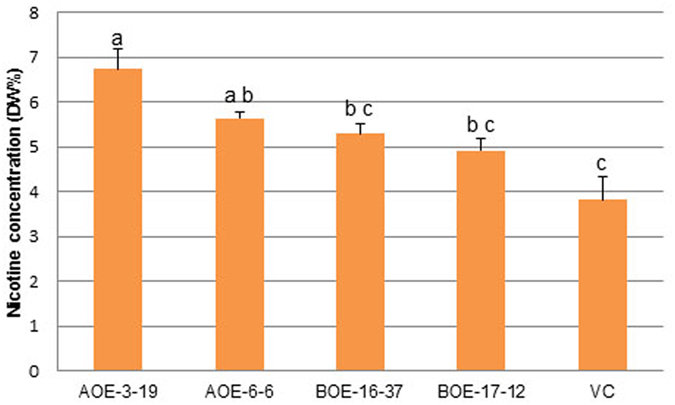
Leaf nicotine levels of T_2_ *NtMYC2* overexpression lines after topping in field. DW%: dry weight percentage. Different letters indicate significant difference (t-test, *P* < 0.05). VC: Vector Control; AOE: *NtMYC2a* overexpression; BOE: *NtMYC2b* overexpression.

To determine the effect of *NtMYC2a* and *NtMYC2b* overexpression on nicotine accumulation under field conditions, and to evaluate the possible effects of overexpression of these genes on overall plant performance, we carried out field experiments to compare vector control, two non-segregating *NtMYC2a* overexpression T_2_ lines (AOE-3–19, AOE-6-6) and two non-segregating *NtMYC2b* overexpression T_2_ lines (BOE-16-37, BOE-17-12). The field-grown transgenic lines exhibited no obvious phenotypic differences from vector control parental line, even though nicotine levels in AOE-3-19 and AOE-6-6 were significantly greater than the vector control (76% and 48% higher, for lines AOE-3-19 and AOE-6-6, respectively) (Student’s t-test, *P* < 0.05). The two *NtMYC2b* overexpression lines did not exhibit a significant increase in nicotine levels (Fig. 6).

**Figure 7:**
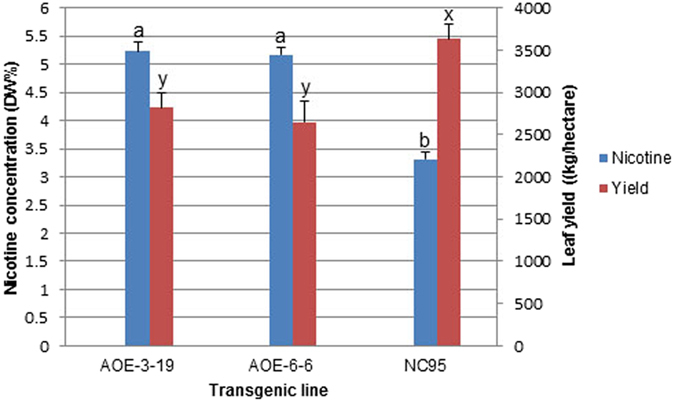
Leaf nicotine levels and yields of T_3_ *NtMYC2a* overexpression lines after topping in field. DW%: dry weight percentage. Different letters indicate significant difference within each category (t-test, *P* < 0.05). VC: Vector Control; AOE: *NtMYC2a* overexpression.

Because the *35S:NtMYC2a* construct exhibited a much greater impact on nicotine levels, we continued to evaluate the T_3_ generation of these *NtMYC2a* overexpression lines in the field. Again, very significant increases in nicotine levels were observed (Fig. 7) (58% and 56% higher, for lines AOE-3-19 and AOE-6-6, respectively). However, the cured leaf yields of these lines were reduced by 23% and 26%, respectively, as compared to the control. Although yields were decreased by about one fourth in both lines, the total nicotine production per hectare for the overexpression lines was still increased by 22% and 14%, respectively.

### The expression of nicotine synthetic genes was inhibited by nicotine

**Figure 8:**
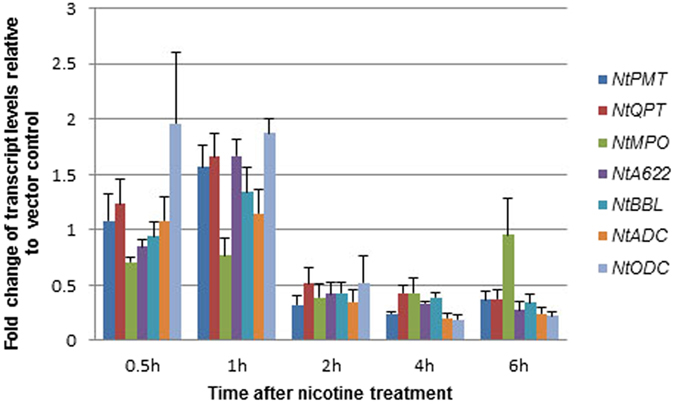
Expression of nicotine synthetic genes affected by exogenously supplied nicotine. The expression level at 0 h was set to 1 and expression at other time points is shown relative to this level. Values are average of three replicates. The error bars indicate standard errors.

Previous studies showed that high levels of nicotine can be toxic to tobacco nicotine-producing root cells, and maintenance of a relatively low cytoplasmic nicotine concentration is necessary for normal root cell functions^26^,^27^. Since the expression of most nicotine synthetic genes in *NtMYC2a* overexpression lines was down regulated, we further tested whether nicotine has a role in regulating the expression of its own synthetic genes. qRT-PCR results showed that the expression levels of all nicotine synthetic genes were repressed to about one-half two hours after root treatment of tobacco seedlings with 0.4 mM exogenously applied nicotine (Fig. 8).

## Discussion

Previous reports have indicated that expression of the major genes involved in nicotine biosynthesis was coordinately regulated to some extent by MeJA treatment^28^,^29^,^30^. These findings prompted us to hypothesize that a major or master transcription factor related to the MeJA signaling pathway might exist to co-regulate the nicotine synthetic genes for nicotine formation. Since overexpression of two key genes in the nicotine biosynthesis pathway (*NtPMT1a* and *NtQPT2)*, individually or simultaneously, did not increase the nicotine content in cultivated tobacco, this leads to the suggestion that other key gene(s) might exist in the pathway and their expression might also need to be altered to substantially increase the nicotine accumulation in the tobacco plant^16^. In the current study, we attempted to identify a putative master transcription factor with the hope that this master transcription factor would regulate all the nicotine synthetic genes, and that manipulation of the expression of this gene would help us achieve the goal of generating transgenic tobacco plants with elevated leaf nicotine accumulation.

Although some transcription factors have been reported to positively regulate nicotine biosynthesis, none of them has been reported to increase the nicotine level in a field-grown, cultivated tobacco variety^17^,^19^,^24^,^31^,^32^,^33^.

To use a plant-based system for secondary metabolite production, greenhouse experiments can provide valuable initial information. Field testing is necessary, however, to determine the effect of a given technology because this is where crop plants are ultimately grown. Positive results in greenhouse environments may not always translate into similar results in actual field growing environments. In this study, we used tobacco cultivar NC95 to evaluate the potential of overexpression of *NtMYC2a or NtMYC2b* gene to increase the nicotine accumulation in field-grown tobacco.

Constitutive overexpression of *NtMYC2a* and *NtMYC2b* was shown to significantly increase leaf nicotine levels in *N. tabacum* in T_0_ and T_1_ generation non-topped or topped greenhouse-grown plants (Figs 3 and 5). Consistent with these results, the T_2_ and T_3_ generations of the *NtMYC2a* overexpression lines exhibited increased nicotine concentrations in field testing, indicating that the high nicotine trait can be stably inherited (Figs 6 and 7). Results from the current study clearly show that *NtMYC2a* is more effective for increasing nicotine content than *NtMYC2b*.

From an industrial perspective, both nicotine concentration and leaf yield are important for nicotine production. In the present study, we observed that the leaf yield of both T_3_ *NtMYC2a* overexpression lines decreased by approximately one-fourth. Indeed, there are reports of negative genetic correlations between yield and nicotine content in non-transgenic populations of *N*. *tabacum*^34^. The negative correlation could be caused by competition for plant resources necessary for both nicotine biosynthesis and tobacco plant growth such as nitrogen. If this is the case, increased nitrogen fertilizer application might partially alleviate the decreases in leaf yields. In our experiments, although leaf yields were decreased, the total nicotine yield per hectare in an AOE3 line was still 22% higher than would have otherwise been achieved using the non-transgenic tobacco control line.

It was expected that the transcript levels of nicotine synthetic genes would increase in the *NtMYC2b* and *NtMYC2a* overexpression T_0_ or T_1_ plants since nicotine levels were significantly increased. Our results indicated, however, that overexpression of *NtMYC2b* in transgenic tobacco plants did not lead to obvious increases in transcript levels of nicotine synthetic genes in root tissues and the expression of all nicotine synthetic genes was remarkably suppressed in *NtMYC2a* overexpression lines (Fig. 4). These observations are similar to a previous report that overexpression of *NbbHLH2* reduced the transcript levels of most nicotine synthetic genes while the nicotine levels were significantly increased in MeJA-treated transgenic plants^17^. Since nicotine was shown to retard tobacco root growth and so to be toxic to the tobacco root cell at relative high concentration^26^, and exogenously supplied nicotine reduced the activity of PMT in tobacco root at the time of decapitation^35^, we speculated that high level of nicotine may inhibit the expression of its own synthetic gene(s) to maintain it a reasonable level in tobacco root to ensure normal root cellular functions. To gain more insights, we performed a nicotine feeding experiment and the data (Fig. 8) showed that nicotine did down regulate the expression of its biosynthetic genes. Thus, a plausible explanation of down regulation of nicotine synthetic genes’ expression in *NtMYC2a* overexpression plants is that, high levels of nicotine produced in the roots of transgenic plants may trigger negative feedback regulation on the expression of its synthetic genes. In a similar case, flavonol-dependent feedback inhibition of transcription of several flavonol synthetic genes appears to prevent the accumulation of toxic non-glycosylated flavonols in *Arabidopsis thaliana*^36^.

To achieve further increases in nicotine accumulation in tobacco leaves, strategies might be pursued to target the process of uploading of nicotine into the xylem and/or translocation and accumulation to leaf vacuoles. Recent research has revealed two vacuole-localized transporters responsible for the sequestering of nicotine into the vacuole of root and leaf (MATE1/2, NtJAT1) and one plasma membrane transporter (NUP1) responsible for importing nicotine from the apoplast into the cytoplasm of tobacco root cells^5^,^26^,^37^. However, transporters responsible for loading of nicotine into the xylem in the root, root to shoot transportation, and unloading of nicotine into leaf cells remain unknown. Characterization of those transporters might be helpful to further enhance nicotine levels in tobacco leaves.

In summary, we isolated two bHLH transcription factor genes, *NtMYC2a* and *NtMYC2b*, in this study. Overexpression of *NtMYC2a* was shown to substantially increase nicotine accumulation in the cultivated tobacco variety NC95. The high nicotine trait was stably inherited to the T_1_, T_2_ and T_3_ generations, and field tests showed that nicotine concentrations reached as high as 6.74% and 5.23% dry weight in tobacco leaves in the T_2_ and T_3_ generations of *NtMYC2*a overexpression lines, levels that were significantly higher than the controls. Together with the leaf yield data of T_3_ lines, as high as a 22% increase in total nicotine production per hectare was achieved in *NtMYC2*a overexpression lines, thus indicating a great potential of *NtMYC2*a overexpression in tobacco plants for industrial nicotine production.

## Methods

### Yeast one-hybrid experiments for cloning transcription factors

A yeast one-hybrid system, Matchmaker^TM^ One-Hybrid Library Construction and Screening Kit (Clontech, Mountain View, CA, USA), was employed to screen for transcription factors that bind to the promoter of the tobacco *NtQPT2* gene. The promoter, 1034 bp in length, was cleaved from construct pTobRD2-PMTOX provided by 22nd Century, LLC (Buffalo, NY, USA), and inserted upstream of the GAL4 minimal promoter in vector pHIS2.1 to form the bait construct pTobHis.

Total RNA was extracted from roots of two-month-old greenhouse-grown tobacco plants (cv. NC95) 0.5 h after topping and was used for cDNA preparation. Screening of positive colonies was performed according to the kit manual. Sequences of the cDNA inserts of positive colonies were used for BLAST analysis with the NCBI GenBank database to identify transcription factors.

### Recovery of full-length cDNAs of the TFs

A GeneRacer kit (Invitrogen, Carlsbad, CA, USA) was used to obtain the missing 5′ sequences of the cDNAs following manufacturer’s instructions. The gene-specific primer used in RACE for *NtMYC2* was

*NtMYC2*GSP: 5′-ACACATTTGGTACAACAGCTCTAAGTGC-3′.

The primers designed to obtain the full-length coding sequences of *NtMYC2*a and *NtMYC2b* are listed in Supplementary Table S1.

All PCR reactions were performed using high-fidelity Taq DNA polymerase (Phusion, Finnzymes, Espoo, Finland). PCR products were cloned into pCR-BluntII-TOPO or pCR4-TOPO vector (Invitrogen) for sequencing analysis.

### Generation of transgenic tobacco plants for overexpression of the *NtMYC2* genes

Binary vector pBI121^38^ was used as the backbone to produce *NtMYC2a* or *NtMYC2b* overexpression constructs. The coding sequences of the *NtMYC2* genes were cleaved from their cloning vectors (pCR-BluntII-TOPO or pCR4-TOPO) and inserted into pBI121 to replace the *GUS* gene.

Transgenic plants were produced from cv. NC95 by using standard *Agrobacterium tumefaciens*-based leaf disc transformation^39^. Initial primary transformants (T_0_) plants were grown in the greenhouse. T_0_ plants were self-pollinated to produce T_1_ generation plants that were tested the presence of transgene using vector specific 5′primer (GTGGCTCCTACAAATGCCATCA) and gene-specific 3′ primers (CAAACACAAGTTGCTTACTGG for *NtMYC2a* and CAAACACAAGTTGCTTACTAC for *NtMYC2b*). T_1_ plants were self-pollinated to produce the T_2_ generation, and non-segregating T_2_ lines were selected on MS medium containing 100 mg/L kanamycin (total 60 seeds, all germinated into healthy seedlings). Plants from non-segregating T_2_ lines were self-pollinated to produce T_3_ generation plants.

For T_0_ and transgenic T_1_ plants grown in greenhouse without topping, leaf samples were collected just before flowering. For transgenic T_1_ plants with topping, leaf samples were collected ten days after topping (plants were topped immediately prior to flowering). From each plant, all leaves were collected, dried at 60 °C for three days and ground for nicotine quantification.

### Tobacco seedlings treated with nicotine

The roots of tobacco plants (cv. Yunyan 87) at the four-leaf stage were immersed in a solution of 0.4 mM nicotine. Roots tissues were collected to investigate expression levels of genes involved in the nicotine biosynthesis pathway (*NtPMT*, *NtQPT*, *NtMPO*, *NtA622*, *NtBBL*, *NtADC* and *NtODC*) at the time points of 0 h, 0.5 h, 1 h, 2 h, 4 h and 6 h post-treatment.

### Northern analysis of *NtMYC2a/b* expression in T_0_ plants

T_0_ tobacco plants were grown in greenhouse for about two months until just before flowering. Around 700 mg root tissue from each plant was used for RNA isolation. Total RNA was extracted by TRIzol (Invitrogen) according to the manufacturer’s manual. The extracted RNA was dissolved in DEPC treated water and quantified by Nanodrop (ND-1000, Thermo Fisher Scientific, Waltham, MA, USA). Ten μg RNA was separated on 1% agarose gel in MOPS buffer. The gel was stained with EtBr and the image was taken under UV. Separated RNA on the gel was blotted onto the Hybond-N+ nylon membrane (Amersham, Arlington Heights, IL, USA).

The primers designed from the coding sequence of *NtMYC2b* for generating the probes for northern hybridization were:

*NtMYC2a*/*b*NOR F: 5′-GAAGTAACGGATACTGAATGG-3′

*NtMYC2*a/*b*NOR R: 5′-ATCCTTGTGTTTGCTGAGAAT-3′

The probe is a 505 bp fragment which shares 94% nt identity to the corresponding region of *NtMYC2a* and thus used to test the transcript level of both NtMYC2 genes (*NtMYC2a*/*b*).

### qRT-PCR analysis of nicotine synthetic genes expression

Root tissue was collected from T_0_ and T_1_ transgenic plants just before flowering. RNA was isolated from root tissue using TRIzol reagent (Invitrogen) according to the manufacturer’s manual and first strand cDNA was synthesized using SuperScript™ III Reverse Transcriptase (Invitrogen) with an oligo (dT) primer. qRT-PCR was performed using FastStart Universal SYBR Green Master (Rox) (Roche, Mannheim, Germany) on ABI7900 (Applied Biosystems, Foster City, CA, USA). The *N. tabacum* actin gene was used as a control for normalization. Three technical replicates were performed using the following PCR program: 50 °C for 1 min; 95 °C for 15 sec; 40 cycles of 95 °C for 15 sec followed by 63 °C for 1 min using the primers for nicotine synthetic genes listed in Supplementary Table S2. For T_0_ plants, two transformation events per gene construct were analyzed. For T_1_ plants, two transgene positive plants from each T_1_ line and total four plants for each *NtMYC2* gene overexpression construct were analyzed. For nicotine-treated tobacco seedlings, three plants from each time point were analyzed. The means and standard errors are presented.

### Field tests of T_2_ and T_3_ generations of the *NtMYC2* overexpression lines

Two *NtMYC2a* and two *NtMYC2b* non-segregating overexpression lines (T_2_ generation) were compared for nicotine levels in a field experiment relative to a vector control. The experimental design was a randomized complete block design with three replications and each plot consisting of 15 plants. Plants were topped immediately prior to flowering. Ten days after topping, the first priming was harvested. The following priming was harvested ten days after the previous one. All the leaves at the stalk position #10 (from the top to the bottom) from the second priming from each replicate were dried, ground, and analyzed for nicotine contents using methodologies described below.

Similar field tests were performed to examine the performance of two T_3_ *NtMYC2a* overexpression lines in a replicated experiment using a randomized complete block design with four replications. Plots consisted of single 20-plant rows. Leaves were harvested in three separate harvests (primings) and flue-cured. Each priming was weighed to generate yield data. Fifty-gram cured leaf samples were prepared for each plot by compositing cured leaf from each priming on a weighted-mean basis for nicotine quantification.

### Quantification of nicotine

Each sample was prepared by placing 0.2000 ± 0.0010 g of dried ground tobacco leaves into a 50 mL Erlenmeyer flask, and 2 mL of 2N NaOH solution was added to each flask and swirled to moisten the tobacco. After 15 min of rest, 10 mL of methyl tertiary butyl ether (MTBE) containing 0.1062 g/mL of quinoline was added to the flask. The flasks were placed on a shaker for 2.5 hrs. After shaking the flasks were allowed to sit overnight to separate. Approximately 1 mL of the top MTBE layer was transferred into a vial. GC analysis was conducted using a split injection (40:1) on an Agilent HP 6890 GC-FID (Agilent Technologies, Santa Clara, CA) using a 30 meter DB-5 MS column (0.53 mm ID and 1.5 μm film thickness). The carrier gas was helium at a linear velocity of approximately 38 cm/sec. The injector and detector were both set at 250 °C. The analysis consists of a temperature program from 110 °C initially held for 0.5 min followed by a ramp to 280 °C at a rate of 25 °C/min where the final temperature was held for 20 min. Data were collected and analyzed using Agilent Chemstation software. A multipoint internal standard calibration table was constructed for nicotine.

### Statistical analysis

Two-tailed Student’s t-tests were performed for comparisons of any two groups within each experiment. Probability values of <0.05 were considered statistically significant.

## Additional Information

**How to cite this article**: Wang, B. *et al*. Genetic Factors for Enhancement of Nicotine Levels in Cultivated Tobacco. *Sci. Rep*. **5**, 17360; doi: 10.1038/srep17360 (2015).

## Supplementary Material

Supplementary Information
