# Supplementary material for: Genetic Factors for Enhancement of Nicotine Levels in Cultivated Tobacco
Source: Sci Rep. 2015 Dec 2;5:17360. doi: 10.1038/srep17360 (PMC4667290; doi:10.1038/srep17360)

Supplementary Information

Title: Genetic Factors for Enhancement of Nicotine Levels in Cultivated Tobacco

Authors: Bingwu Wang[1.2*], Ramsey S. Lewis[2], Junli Shi[1], Zhongbang Song[1], Yulong Gao[1], Wenzheng Li[1], Hongxia Chen[2] and Rongda Qu[2]

[1]Tobacco Breeding Center, Yunnan Academy of Tobacco Agricultural Sciences, Kunming, Yunnan 650021, China

[2]Crop Science Department, North Carolina State University, Raleigh, NC 27695, USA

*Corresponding author:

Bingwu Wang

Email: bwwang76@hotmail.com

Tel: +86-877-2075112

Fax: +86-871-65101149

Supplementary Table S1. List of primers used to obtain full-length *NtMYC2a* and *NtMYC2b* sequences.

Gene name	Forward (F) or Reverse (R)	Sequence from 5'-3'
NtMYC2a	F	GCGGTCTAGACAGATCTGAATTGATTTGTCT
NtMYC2a	R	GCGGTCTAGAACATTATTCAGAGCTCACTATG
NtMYC2b	F	GCGTCTAGAATGACGGACTATAGAATACCA
NtMYC2b	R	GCGTCTAGATCATCGCGATTCAGCAATTCT

Supplementary Table S2. List of primers used for qRT-PCR analysis.

Gene name	Forward (F) or Reverse (R)	Sequence from 5'-3'
NtPMT	F	GCTATTATAGTGGACTCTTCTG
NtPMT	*R*	TGTGTGCATACAACTCCTCCT
NtQPT	*F*	GTTGAGGTTGAAACCAGGAC
NtQPT	*R*	GAACAACCATATTGTCCAGCA
NtMPO	*F*	CCTACTAAGCTTCCTCCAAG
NtMPO	*R*	TCGAGCAGCAGCATGTACTT
NtODC	*F*	CTTGTGATGCTCTTGATACTG
NtODC	*R*	GCGAGGTGAGTAACAATGG
NtADC	*F*	GTGTCTGCTTCTAGTCACTC
NtADC	*R*	TACTCTCCACGAACTGCAGC
NtA622	*F*	GGATGATAGAGGCAGAAGGA
NtA622	*R*	TGACAACTTTGTCTCTAGGAG
NtNBBL	*F*	GTATCTAG CACAGTGGAATG
NtNBBL	*R*	CAAAGGGCGTCATTGTATTG
Actin	*F*	CTGAGGTCCTTTTCCAACCA
Actin	*R*	TACCCGGGAACATGGTAGAG

Supplementary Fig. S1. Nicotine biosynthesis pathway. Solid arrows indicate steps catalyzed by known enzymes; dashed arrows indicate ill-defined steps.

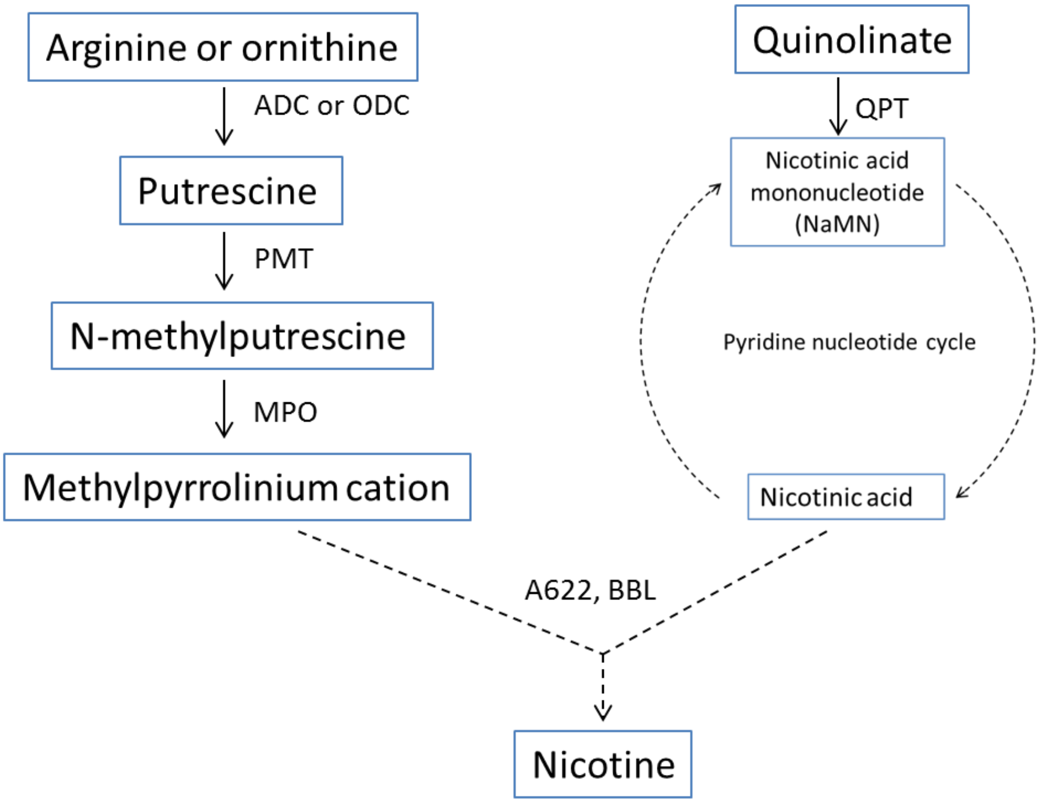

Supplement: Supplementary Information [file srep17360-s1.pdf]
